# Combining IMWG GA and hematopoietic score to optimize the evaluation of dynamic chemotherapy tolerance in multiple myeloma

**DOI:** 10.1080/07853890.2025.2558127

**Published:** 2025-10-14

**Authors:** Yadong Chen, Fenfen He, Yanyu Chen, Zhibin He, Dongxia Wu, Huihua An, Qian Liu, Yongjiang Zheng

**Affiliations:** a Department of Hematology, The Third Affiliated Hospital of Sun Yat-sen University, Guangzhou, China; b Functional Department, Southern Theater Command Air Force Hospital, Guangzhou, China; c Department of Hematology, The Third Affiliated Hospital of Sun Yat-sen University Zhaoqing Hospital, Zhaoqing, China

**Keywords:** Multiple myeloma, chemotherapy tolerance, geriatric assessment, hematopoietic score, risk stratification

## Abstract

**Background:**

Chemotherapy tolerance significantly affects treatment efficacy and survival in multiple myeloma (MM), especially among frail patients. The International Myeloma Working Group Geriatric Assessment (IMWG GA) is widely used for frailty assessment, but limitations remain. Dynamic and integrated assessment approaches may improve risk stratification and support personalized treatment.

**Methods:**

We retrospectively analyzed 111 newly diagnosed MM patients treated at the Third Affiliated Hospital of Sun Yat-sen University (Jan 2018–Jun 2024). IMWG GA and Hematopoietic Score (HS) were assessed at baseline and follow-up. Static and dynamic frailty assessments were compared. Chemotherapy-related adverse events (AEs) were graded per CTCAE 5.0.

**Results:**

Chemotherapy tolerance and frailty status changed across cycles. Static assessments showed limited predictive value, while dynamic evaluation with IMWG GA and HS improved risk stratification. IMWG GA differentiated Fit vs. Frail patients for total AEs (HR = 1.81, *p* = 0.001) and non-hematological AEs (HR = 1.90, *p* = 0.002); HS strongly stratified hematological AEs (HR=9.91, *p* < 0.001). Combining both tools, the Hemo-IMWG GA model achieved superior predictive performance for total toxicity (AUC 0.619–0.646; C-index 0.615) and non-hematological toxicity (AUC 0.600–0.618; C-index 0.605), and improved stratification of hematological toxicity (C-index 0.687 vs. 0.600 for IMWG GA, though slightly below HS alone, C-index 0.717).

**Conclusion:**

Hemo-IMWG GA offers a dynamic, integrated approach for evaluating chemotherapy tolerance in MM. By combining functional and hematopoietic dimensions, it improves prediction of treatment-related toxicity—especially hematological AEs—and aids personalized treatment decisions.

## 1. Introduction

The introduction of novel therapies and autologous hematopoietic stem cell transplantation has significantly improved outcomes for multiple myeloma (MM) patients, with some surviving more than ten years [1]. However, poor chemotherapy tolerance remains the key factor limiting treatment efficacy and long-term survival. Frail patients, with physical weakness and reduced overall health, are more susceptible to chemotherapy-related toxicities, increasing the risk of early mortality and reducing treatment efficacy due to therapy discontinuation or dose reductions [2]. Early identification of frail patients and appropriate management is essential for improving prognosis.

The Geriatric Assessment (GA) by the International Myeloma Working Group (IMWG) is the most widely used tool for frailty evaluation in MM patients. While valuable for frailty assessment, the IMWG GA has limitations, especially in distinguishing hematological toxicity risks [3,4]. Although IMWG GA has been used for dynamic assessments [5], indicators such as age, Instrumental Activities of Daily Living (IADL), and Charlson Comorbidity Index (CCI) show minimal changes during short-term treatment, limiting its ability to track dynamic shifts in frailty.

Routine blood tests are commonly used to monitor disease status, treatment responses, and overall health [6]. The Hematopoietic Score (HS), proposed by AlSaleh et al., is based on hemoglobin (Hb), mean corpuscular volume (MCV), and platelet count (PLT) and is an independent prognostic factor for MM patients [7]. Good hematopoietic function is essential for immune health, and poor hematopoiesis increases the risk of infections [8,9]. Hb levels correlate with physical performance and frailty status, MCV reflects nutritional levels and hematopoietic regulation in the bone marrow, and PLT decline is associated with bone marrow function deterioration due to tumor burden and disease progression [10–12]. Our previous research has demonstrated that physical performance and nutritional status are key factors influencing chemotherapy tolerance [4]. Based on these findings, we propose that HS is linked to chemotherapy tolerance in MM patients and could serve as a dynamic assessment tool.

This study aims to evaluate the utility of IMWG GA and HS in dynamic chemotherapy tolerance assessment and combined risk stratification, with the goal of improving the accuracy and clinical applicability of these assessments to support personalized treatment strategies for MM patients.

## 2. Methods

### 2.1. Study design and patient characteristics

This study is a single-center, retrospective analysis of newly diagnosed MM (NDMM) patients at the Third Affiliated Hospital of Sun Yat-sen University between January 2018 and June 2024. The study was conducted in accordance with the principles outlined in the Declaration of Helsinki and was approved by the hospital’s ethics committee (approval number: RG2025-013-01). All patients were adults aged 18 years or older. A standardized broad informed consent form was signed by each patient upon initial hospital admission. For patients who were unable to provide consent due to cognitive or physical conditions, consent was obtained from a legally authorized representative. The broad informed consent covered participation in treatment procedures, reporting of adverse events (AEs), and the use of anonymized clinical and laboratory data for research purposes, in accordance with institutional and ethical guidelines.

Patients were eligible for inclusion if they met the 2014 IMWG diagnostic criteria for MM, agreed to undergo chemotherapy, and received at least two cycles of anti-myeloma therapy. Additional inclusion criteria included the availability of complete baseline and follow-up data—such as hematological test results, IMWG GA assessments, and documentation of grade ≥3 AEs—as well as adherence to scheduled follow-up visits. Patients were excluded if they refused chemotherapy, had missing baseline or AE data, or were unable to complete follow-up.

High-risk cytogenetic abnormalities were assessed using pre-treatment fluorescence *in situ* hybridization (FISH) and conventional karyotyping, following the 2016 IMWG consensus criteria [13], including translocations t(4;14), t(14;16), t(14;20), del(17/17p), gain(1q), non-hyperdiploid karyotype, and del(13). The Second Revision of the International Staging System (R2-ISS) was used to assess MM tumor burden. For cases with partially missing cytogenetic data, we followed the approach described in previous studies [14]: patients were classified as R2-ISS stage III or IV when the cumulative score from available data was ≥1.5 or ≥3.0 points, respectively. Cases not meeting these criteria were labeled as “Not classified.”

### 2.2. IMWG GA and HS assessment

IMWG GA and HS data were collected at baseline and follow-up for dynamic frailty and chemotherapy tolerance assessment.

IMWG GA was performed using four components: age was categorized as <75 years (0 points), 75–80 years (1 point), and >80 years (2 points). ADL and IADL were evaluated using the Katz ADL and the Lawton IADL Scale, respectively. A score of ≤4 for ADL or ≤5 for IADL indicated dependency and was assigned 1 point each, while independence in either was assigned 0 points. A Charlson Comorbidity Index (CCI) >1 was assigned 1 point; a score of 0–1 received 0 points. Based on the total score (0–5), patients were classified as Fit (0 points), Intermediate Fit (Int-Fit; 1 point), or Frail (2–5 points) according to the IMWG GA criteria [3].

HS assessment is based on three criteria: Hb ≤100 g/L, MCV ≥96 fL, and PLT ≤150 × 10^9^/L. Each criterion contributes one point, with a total score of 0–3 points. Patients were classified into three groups: Fit (0 points), Int-Fit (1 point), and Frail (2–3 points). Patients received routine blood tests before each chemotherapy cycle, with the HS score calculated from the most recent pre-treatment blood count.

### 2.3. Static vs. dynamic frailty assessments

Static and dynamic frailty assessments were conducted to evaluate frailty status. The static assessment was a one-time evaluation carried out before the first chemotherapy cycle and remained unchanged throughout the induction period.

The dynamic assessment was conducted at predefined time points prior to each chemotherapy cycle. When multiple updates of relevant laboratory or clinical indicators were available for a given cycle, the assessment closest to the scheduled chemotherapy date was selected for analysis. Evaluations of HS and IMWG GA were recalculated based on these data, and frailty risk stratification was updated accordingly for each cycle.

### 2.4. Induction therapy regimens and assessment of chemotherapy tolerance

The initial induction therapies primarily included triplet regimens: (1) a proteasome inhibitor (PI; bortezomib, ixazomib, or carfilzomib) with doxorubicin and dexamethasone (Dex); (2) a PI with an immunomodulatory drug (IMiD; lenalidomide or pomalidomide) and Dex; and (3) daratumumab-based triplet regimens (e.g. with a PI and Dex or with an IMiD and Dex). Additionally, a subset of patients received doublet regimens, most commonly PI + Dex or IMiD + Dex.

Adverse events (AEs) during induction therapy were classified as hematological or non-hematological toxicities and graded according to the Common Terminology Criteria for Adverse Events (CTCAE) version 5.0. Due to the systematic underreporting of grade 1–2 toxicities in retrospective medical records, which likely underestimates the burden of mild toxicities, only grade ≥3 AEs were analyzed to minimize bias and improve the stability and comparability of the results. All grade ≥3 AEs were comprehensively recorded to capture the overall toxicity burden and for recurrent event analysis. For cumulative risk estimation, a per-cycle primary event approach was adopted to avoid attributional ambiguity and double counting from concurrent events; under this approach, only the most clinically significant or clearly treatment-attributable AE was retained per cycle.

### 2.5. Statistical analysis

All statistical analyses were conducted using Python version 3.12. Data visualizations were generated with the Matplotlib and Seaborn libraries. Continuous variables were reported as medians with interquartile ranges or means ± standard deviations, while categorical variables were summarized as counts and percentages.

The Kaplan–Meier method was used to estimate cumulative incidence curves based on per-cycle primary events, and group differences were assessed using the log-rank test. The overall burden of recurrent grade ≥3 toxicities was evaluated using an Andersen–Gill extension of the Cox model, which incorporated all observed grade ≥3 events across cycles, adjusting for age, R2-ISS stage, and chemotherapy regimen.

Model performance was evaluated using the area under the receiver operating characteristic curve (AUC) and Harrell’s C-index with 1,000 bootstrap resamples for internal validation. Net reclassification improvement (NRI) and decision curve analysis (DCA) were additionally performed to assess reclassification and clinical utility. All statistical tests were two-sided, and a P value of < 0.05 was considered statistically significant.

## 3. Results

### 3.1. Baseline characteristics and chemotherapy-related AEs

After applying inclusion and exclusion criteria, 111 MM patients were analyzed. The median age was 61 years (IQR: 55–68), with 23 patients (20.72%) over 70 years and 7 (6.31%) over 75 years. Most patients had a secondary school education or lower (81.98%, 91 patients). 34.23% of patients (38) had high-risk genetic abnormalities. According to the R2-ISS system, 6 patients (5.41%) were in stage I, 31 (27.93%) in stage II, 59 (53.15%) in stage III, and 9 (8.11%) in stage IV, while 6 patients (5.41%) were not classified. A total of 98 patients (88.29%) received a triplet induction regimen. Specifically, 62 patients (55.9%) were treated with PI + doxorubicin + Dex, 27 patients (24.3%) with PI + IMiD + Dex, and 9 patients (8.1%) with daratumumab-based triplets (5 patients with daratumumab + PI + Dex [4.5%], and 4 patients with daratumumab + IMiD + Dex [3.6%]). Additionally, 13 patients (11.7%) received a doublet regimen, including PI + Dex (11 patients, 9.9%) and IMiD + Dex (2 patients, 1.8%). The characteristics are detailed in Table 1.

**Table 1.** Baseline clinical characteristics of 111 newly diagnosed MM patients.

Variable	Category	N (%)	Median (Q1-Q3)
Age (years)			61 (55–68)
	0-60	47 (42.34)	
	60–70	41 (36.94)	
	≥70	23 (20.72)	
Sex	Female	63 (56.76)	
	Male	48 (43.24)	
Education Level	Secondary or Lower	91 (81.98)	
	Tertiary or Higher	20 (18.02)	
Marital Status	Married	107 (96.40)	
	Unmarried	4 (3.60)	
M-Protein Type	IgG	62 (55.86)	
	LC	22 (19.82)	
	IgA	21 (18.92)	
	Others	6 (5.41)	
Cr (µmol/L)			79.0 (59.5–119.5)
	0–90	66 (59.46)	
	≥90	45 (40.54)	
Hb (g/L)			91.0 (73.5–110.0)
	0–90	50 (45.05)	
	90–120	43 (38.74)	
	≥120	18 (16.22)	
LDH (U/L)			171.0 (140.0–241.0)
	0–250	86 (77.48)	
	≥250	25 (22.52)	
ALB (g/L)			32.5 (27.9–38.6)
	≥35	42 (37.84)	
	<35	69 (62.16)	
β₂MG (mg/L)			4.9 (3.2–8.4)
	0-3.5	33 (29.73)	
	≥3.5	78 (70.27)	
High-Risk Cytogenetic	No	49 (44.14)	
	Yes	45 (40.54)	
	Missing	17 (15.32)	
R2-ISS Stage	I	6 (5.41)	
	II	31 (27.93)	
	III	59 (53.15)	
	IV	9 (8.11)	
	Not classified	6 (5.41)	
Chemotherapy Regimens	Doublet Regimens	13 (11.71)	
	*PI + Dex*	11 (9.91)	
	*IMiDs + Dex*	2 (1.80)	
	Triplet Regimens	98 (88.29)	
	*PI + Dox + Dex*	62 (55.86)	
	*PI + IMiDs + Dex*	27 (24.32)	
	*Dara + PI/IMiDs + Dex*	9 (8.11)	

PI, proteasome inhibitor; Dex, dexamethasone; IMiDs, immunomodulatory drugs; Dox, doxorubicin; Dara, daratumumab.

Based on the IMWG GA, 46 patients (41.44%) were classified as Fit, 24 patients (21.62%) as Int-Fit, and 41 patients (36. 94%) as Frail. Based on the HS, 30 patients (27.03%) were classified as Fit, 39 patients (35.14%) as Int-Fit, and 42 patients (37.84%) as Frail (Table 2).

**Table 2.** Distribution of IMWG GA and HS components.

Variable	Category	N (%)
Age	0–75	104 (93.69)
	76–80	3 (2.70)
	≥80	4 (3.60)
ADL	≤4	37 (33.33)
	>4	74 (66.67)
IADL	≤5	45 (40.54)
	>5	66 (59.46)
CCI	≤1	78 (70.27)
	>1	33 (29.73)
IMWG GA	0	46 (41.44)
	1	24 (21.62)
	2–5	41 (36.94)
Hb (g/L)	≤100	67 (60.36)
	>100	44 (39.64)
MCV (fL)	<96	76 (68.47)
	≥96	35 (31.53)
PLT (× 10^9^/L)	>150	77 (69.37)
	≤150	34 (30.63)
HS	0	30 (27.03)
	1	39 (35.14)
	2–3	42 (37.84)

All patients underwent induction therapy with a median of 5 cycles (range: 5–6), totaling 543 cycles. During these cycles, 89 patients(80.18%) experienced a total of 309 grade ≥3 AEs across 195 chemotherapy cycles (Supplementary Table S1) comprising 210 non-hematological (67.96%) and 99 hematological events (32.04%). Among these, 39 grade 4 AEs occurred in 26 patients (23.42%), resulting in ICU admission for 7 patients and 2 treatment-related deaths.

### 3.2. Limitations of static evaluation in predicting chemotherapy-related AEs using IMWG GA and HS

**Figure 1.**
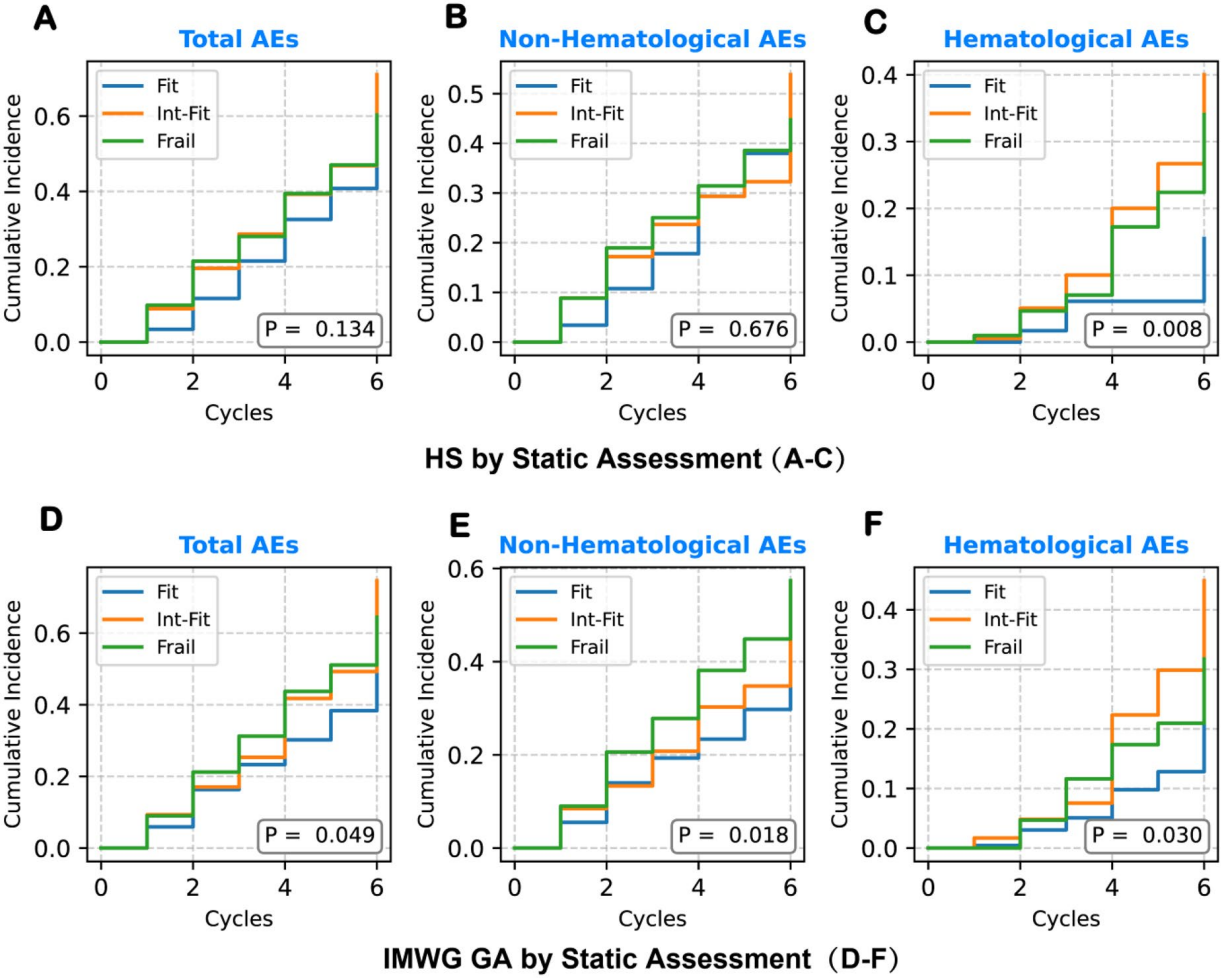
KM curves for chemotherapy tolerance based on static assessment. KM curves depict the cumulative incidence of grade ≥ 3 AEs first major AEs across frailty subgroups (Fit, Int-Fit, Frail) under static assessments: (A–C) using HS for total, non-hematological, and hematological AEs; (D–F) using IMWG GA for corresponding categories. HS showed clear stratification for hematological AEs (C, P < 0.05), but no significant differences for other AE types. IMWG GA demonstrated limited discrimination for total and non-hematological AEs (D, E), but in hematological AEs (F), the Int-Fit group paradoxically exhibited higher risk than the Frail group, limiting its clinical interpretability.

Static evaluations with IMWG GA and HS were performed, and KM curves of AEs during induction chemotherapy were generated (Figure 1). After adjustment for age, R2-ISS stage, and treatment regimen, IMWG GA demonstrated a significant stratification ability for non-hematological grade ≥3 AEs between the Fit and Int-Fit groups (HR = 1.74, 95% CI: 1.14–2.66, *p* = 0.011) (Table 3). However, the Fit vs Frail comparison was not statistically significant across all AE categories (*p* > 0.05). This finding appears counterintuitive, as the risk stratification between the Fit and Frail groups would generally be expected to be more distinct than that between the Fit and Int-Fit groups.

**Table 3.** Risk stratification ability of static IMWG GA and HS for grade ≥3 AEs.

Frailty Model	Subgroup	Total AEs		Non-Hematological AEs		Hematological AEs	
		HR (95% CI)	P	HR (95% CI)	P	HR (95% CI)	P
HS	Fit vs Int-Fit	1.27 (0.88–1.83)	0.200	0.92 (0.62–1.36)	0.676	3.91 (1.51–10.15)	0.005
	Fit vs Frail	1.06 (0.72–1.58)	0.757	0.80 (0.52–1.23)	0.304	5.56 (2.11–14.66)	<0.001
	Int-Fit vs Frail	0.82 (0.59–1.15)	0.260	0.85 (0.57–1.25)	0.402	1.26 (0.75–2.12)	0.390
IMWG GA	Fit vs Int-Fit	1.81 (1.25–2.61)	0.002	1.74 (1.14–2.66)	0.011	2.52 (1.36–4.67)	0.003
	Fit vs Frail	1.37 (0.96–1.96)	0.080	1.48 (1.00–2.20)	0.050	1.50 (0.79–2.87)	0.215
	Int-Fit vs Frail	0.85 (0.59–1.22)	0.381	1.03 (0.68–1.56)	0.877	0.53 (0.29–0.97)	0.039

Adjusted for Age, R2-ISS Stage, and Chemotherapy Regimens.

In contrast, HS significantly stratified hematological grade ≥3 AEs between the Fit and Int-Fit groups (HR = 3.91, 95% CI: 1.51–10.15, *p* = 0.005) and between the Fit and Frail groups (HR = 5.56, 95% CI: 2.11–14.66, *p* < 0.001), while no significant differences were observed in other AE categories. These results underscore the limitations of using a single tool for static predictions of chemotherapy tolerance, highlighting the need for enhanced risk stratification and clinical utility.

### 3.3. Dynamic changes in chemotherapy-related AEs and frailty scores

**Figure 2.**
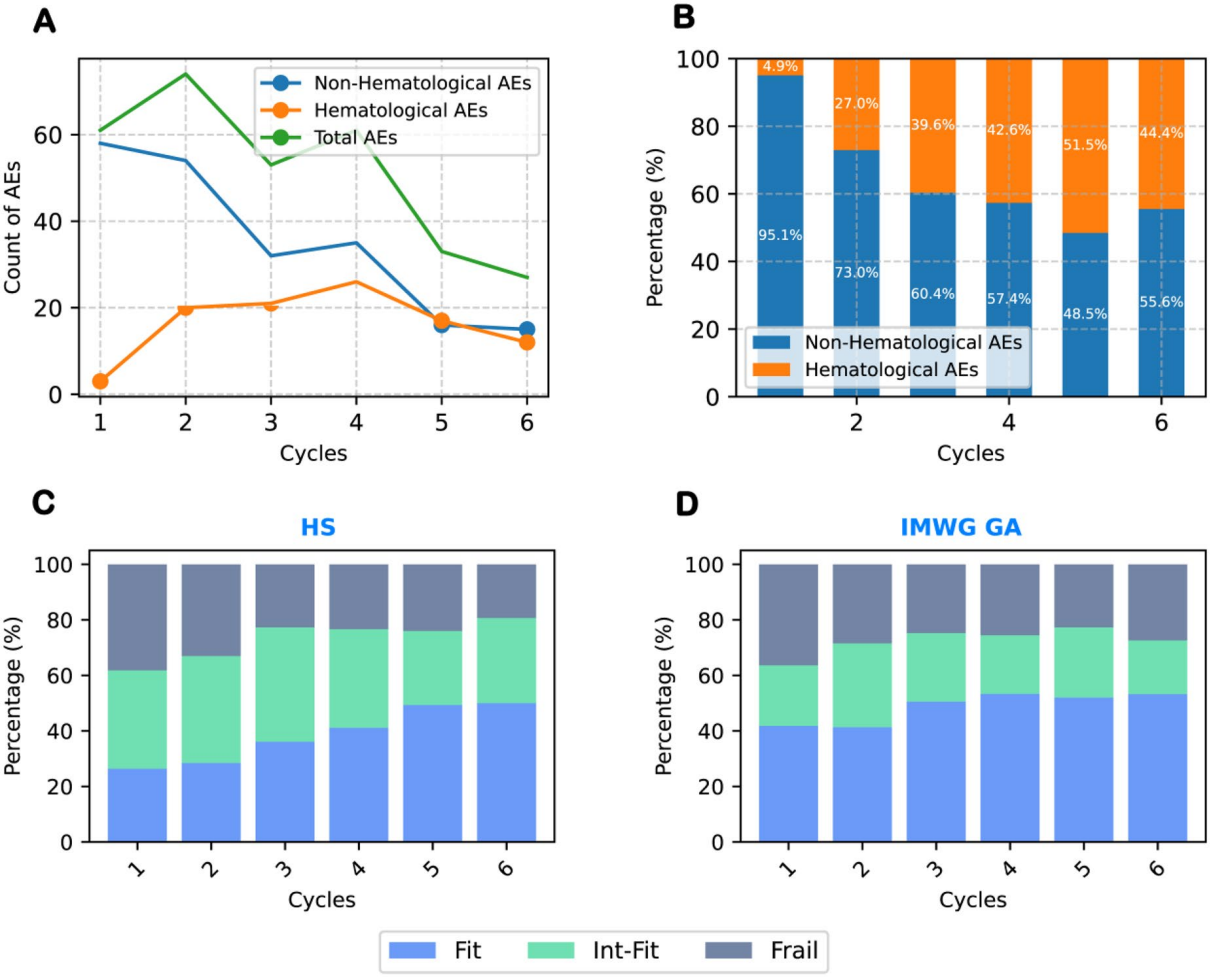
Dynamic changes in AEs and frailty scores across treatment cycles. The incidence of grade ≥ 3 AEs changed dynamically over treatment cycles, as illustrated by the temporal trends in different AE types (A). The total AE rate peaked at cycle 2 and then gradually declined. A more detailed view of the proportional composition of AE types is shown in the stacked bar chart (B): non-hematological AEs predominated in the early cycles, while hematological AEs accounted for an increasing proportion in later cycles. The dynamic distribution of frailty subgroups assessed by HS and IMWG GA is presented in (C) and (D), respectively. Overall, there was a trend toward increased Fit and decreased Frail classifications across treatment cycles. Compared with IMWG GA, which exhibited delayed responsiveness, HS showed greater flexibility in capturing short-term changes in patient status. These findings underscore the importance of dynamic frailty assessment throughout the treatment process.

Considering the limitations of static assessments with IMWG GA and HS, we investigated dynamic changes in chemotherapy-related AEs and score group trends (Supplementary Tables S2 and S3, Figure 2). During induction, AE incidence initially rose from 55.0% to 66.7% by the second cycle, then fell to 24.3% by the sixth cycle (Figure 2A). AE types varied significantly, with early cycles dominated by non-hematological AEs (95.1% of total), gradually decreasing to 48.5% in the fifth cycle, while hematological AEs increased from 4.9% to 51.5% (Figure 2B). This trend shows hematological AEs as the predominant factor affecting chemotherapy tolerance in later stages. Additionally, dynamic shifts in IMWG GA and HS scores during treatment showed an increasing proportion of patients classified as Fit and a decreasing proportion as Frail, particularly between the second and third cycles (Figure 2C and D). HS demonstrated greater flexibility than IMWG GA in dynamic adjustments, particularly in later cycles, indicating its potential superiority in dynamically assessing frailty changes.

From these results, we propose two approaches to optimize frailty status assessment: firstly, by dynamically monitoring changes during chemotherapy to quickly adjust treatment strategies; and secondly, by improving recognition of hematological AEs within the IMWG GA framework to better manage the rising trend of such AEs in later treatment stages, thus enabling more precise patient management.

### 3.4. Dynamic risk stratification in predicting chemotherapy-related AEs using IMWG GA and HS

**Figure 3.**
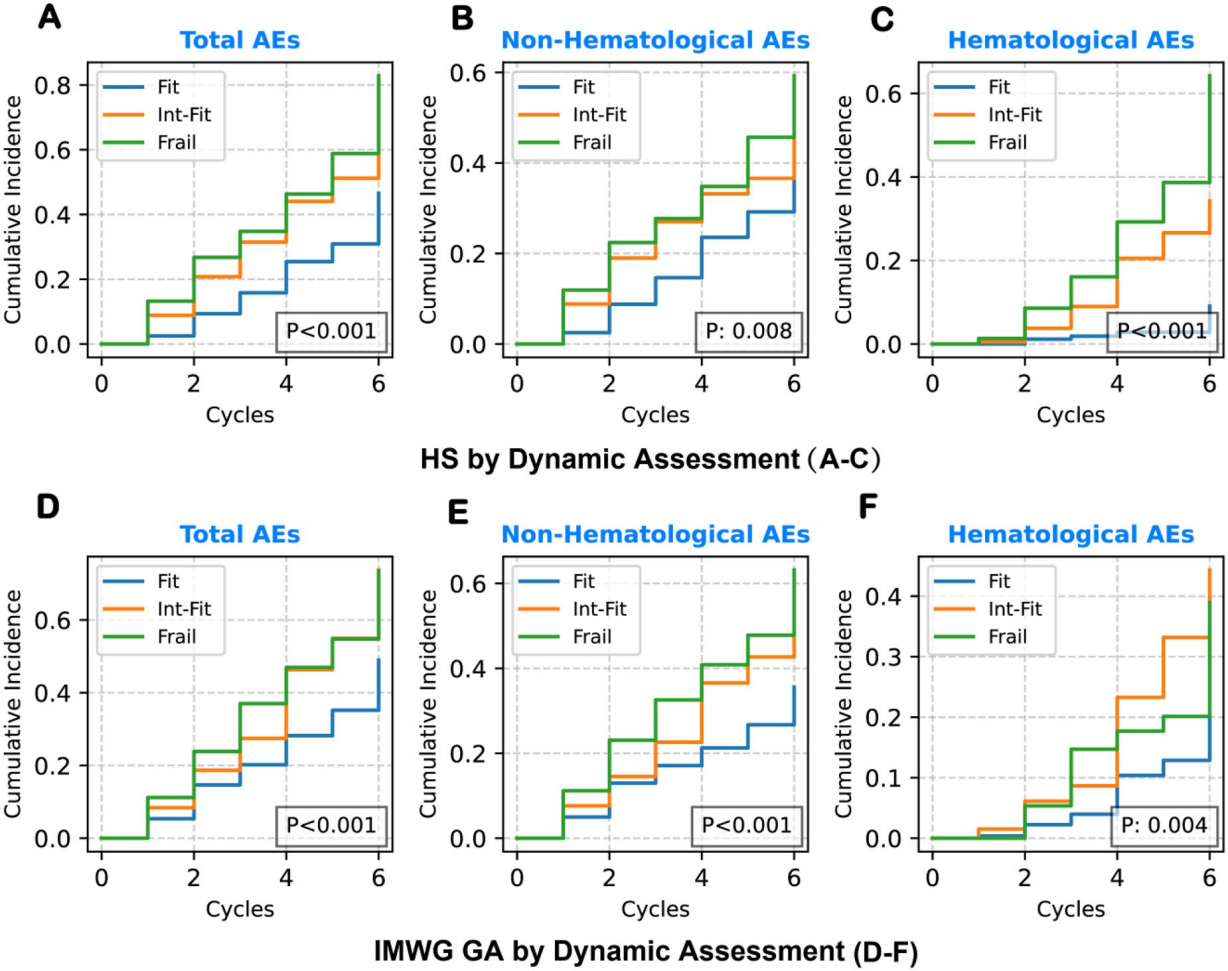
KM curves for chemotherapy tolerance based on dynamic assessment. KM curves depict the cumulative incidence of first major AEs across frailty subgroups (Fit, Int-Fit, Frail) under dynamic assessments: (A–C) using HS for total, non-hematological, and hematological AEs; (D–F) for IMWG GA. Compared with static assessment in Figure 1, dynamic assessment improved risk stratification: HS addressed the previous lack of discrimination for total and non-hematological AEs (A, B), and demonstrated clearer separation between Fit and Frail groups for hematological AEs (C). IMWG GA showed improved discrimination for total and non-hematological AEs (D, E); however, its improvement in hematological AEs (F) remained limited. In addition, the Int-Fit group exhibited substantial variability, occasionally exceeding the Frail group in risk, further limiting its clinical interpretability.

We further employed IMWG GA and HS for dynamic chemotherapy tolerance assessment and risk stratification. Our findings demonstrate a significant improvement in their ability to evaluate chemotherapy tolerance (Figure 3, Table 4). With dynamic assessment applied, IMWG GA effectively stratified risk between the Fit and Frail groups for total AEs (HR = 1.81, 95% CI: 1.26–2.60, *p* = 0.001), non-hematological AEs (HR = 1.90, 95% CI: 1.27–2.84, *p* = 0.002), and hematological grade ≥3 AEs (HR = 2.00, 95% CI: 1.05–3.80, *p* = 0.034). Moreover, HS also effectively stratified risk for total grade ≥3 AEs between the Fit and Frail groups (HR = 1.88, 95% CI: 1.29–2.74, *p* = 0.001) and hematological grade ≥3 AEs (HR = 9.91, 95% CI: 4.07–24.16, *p* < 0.001). However, HS did not significantly stratify non-hematological AEs (*p* > 0.05). These findings highlight that IMWG GA was most effective at stratifying non-hematological grade ≥3 AEs, while HS excelled in stratifying hematological AEs.

**Table 4.** Comparison of dynamic HS, IMWG GA, and Hemo-IMWG GA in grade ≥3 AEs.

Frailty Model	Subgroup	Total AEs		Non-Hematological AEs		Hematological AEs	
		HR (95% CI)	P	HR (95% CI)	P	HR (95% CI)	P
HS	Fit vs Int-Fit	1.51 (1.05–2.17)	0.025	1.12 (0.75–1.66)	0.578	5.05 (2.05–12.41)	<0.001
	Fit vs Frail	1.88 (1.29–2.74)	0.001	1.30 (0.86–1.96)	0.220	9.91 (4.07–24.16)	<0.001
	Int-Fit vs Frail	1.19 (0.85–1.66)	0.303	1.08 (0.73–1.59)	0.708	1.94 (1.15–3.26)	0.013
IMWG GA	Fit vs Int-Fit	1.68 (1.16–2.45)	0.006	1.60 (1.04–2.47)	0.033	2.43 (1.29–4.57)	0.006
	Fit vs Frail	1.81 (1.26–2.60)	0.001	1.90 (1.27–2.84)	0.002	2.00 (1.05–3.80)	0.034
	Int-Fit vs Frail	1.19 (0.82–1.72)	0.364	1.37 (0.91–2.08)	0.133	0.88 (0.47–1.62)	0.673
Hemo-IMWG GA	Fit vs Int-Fit	1.99 (1.38–2.87)	<0.001	1.70 (1.12–2.56)	0.012	3.62 (1.77–7.42)	<0.001
	Fit vs Frail	2.24 (1.55–3.23)	<0.001	1.89 (1.25–2.84)	0.002	5.12 (2.57–10.21)	<0.001
	Int-fit vs Frail	1.14 (0.81–1.60)	0.468	1.13 (0.77–1.67)	0.535	1.33 (0.77–2.30)	0.310

Adjusted for age, R2-ISS stage, and chemotherapy regimens.

Additionally, the Int-Fit group of HS exhibited stratification patterns similar to the Frail group for total grade ≥3 AEs (*p* > 0.05), while both groups were distinctly differentiated from the Fit group (*p* < 0.05). For hematological AEs, HS demonstrated statistically significant risk separation between the Fit and Int-Fit groups (HR = 5.05, 95% CI: 2.05–12.41, *p* < 0.001) as well as between the Int-Fit and Frail groups (HR = 1.94, 95% CI: 1.15–3.26, *p* = 0.013).

In contrast, the AE incidence rate in the IMWG GA Int-Fit group did not consistently align with either the Fit or Frail groups, exhibiting considerable variability and even leading to a higher cumulative incidence of hematological toxicities compared to the Frail group (Figure 3D–F). This instability complicates the interpretation and reduces the clinical utility of the Int-Fit category.

### 3.5. Combining IMWG GA with HS for better chemotherapy tolerance stratification

**Figure 4.**
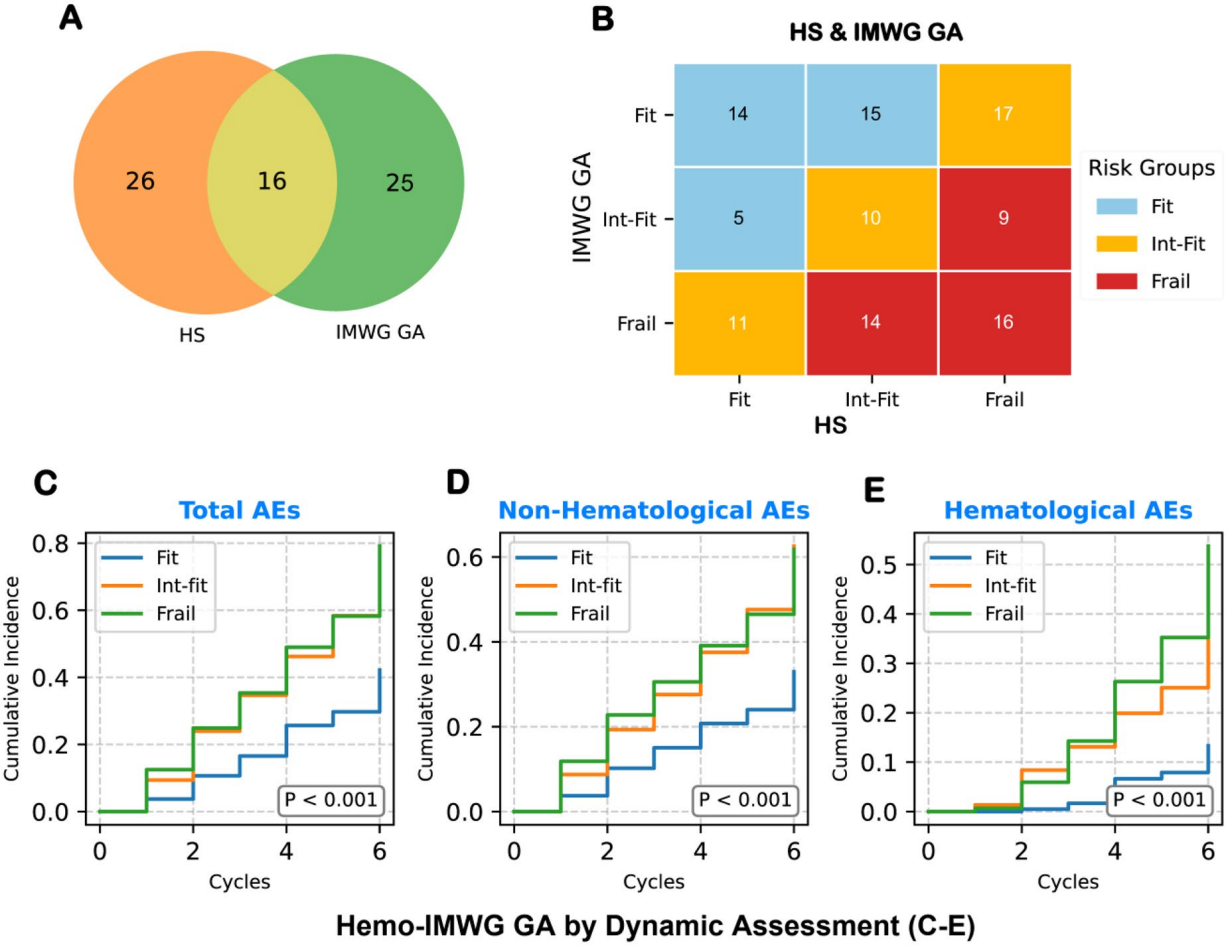
Optimized stratification of chemotherapy tolerance by integrating HS and IMWG GA. Venn diagram (A) shows limited overlap between the HS high-risk group and the IMWG GA Frail group (39.02%), suggesting strong complementarity. Matrix heatmap (B) illustrates the construction of the Hemo-IMWG GA scoring system, which integrates HS into the IMWG GA framework to refine frailty classification. Each tool was assigned a score of 0 (Fit), 1 (Int-Fit), or 2 (Frail); their sum (range: 0–4) defined the combined categories: Fit (score 0–1, blue), Int-Fit (score 2, orange), and Frail (score 3–4, red). The number of patients in each cell is shown. (C–E) KM curves depict the cumulative incidence of first major AEs across Hemo-IMWG GA subgroups for total, non-hematological, and hematological AEs, respectively. The integrated model markedly improves separation between Fit and Frail groups for total and non-hematological AEs, maintains robust stratification for hematological AEs, and shifts Int-Fit patients closer to Frail across all AE types, thereby enhancing interpretability and clinical utility.

We combined IMWG GA with HS to enhance chemotherapy tolerance identification accuracy, analyzing their complementarity. A Venn diagram (Figure 4A) indicated minimal overlap between the HS high-risk group and the IMWG GA Frail group (39. 02%, 16 patients), suggesting strong complementarity. Subsequently, IMWG GA and HS were each assigned a score of 0 (Fit), 1 (Int-Fit), or 2 (Frail), and the sum of the two (range: 0–4) was used to define the combined Hemo-IMWG GA subgroups: Fit (score 0–1), Int-Fit (score 2), and Frail (score 3–4) (Figure 4B).

Further analysis showed that the Hemo-IMWG GA outperformed IMWG GA (Figure 4C–E, Table 4). Specifically, Hemo-IMWG GA significantly improved risk stratification between Fit and Frail groups for both total grade ≥3 AEs (HR = 2.24, 95% CI: 1.55–3.23, *p* < 0.001) and non-hematological grade ≥3 AEs (HR = 1.89, 95% CI: 1.25–2.84, *p* = 0.002). For hematological grade ≥3 AEs, Hemo-IMWG GA continued to show superior stratification (HR = 5.12, 95% CI: 2.57–10.21, *p* < 0.001).

Additionally, the Hemo-IMWG GA similarly stratified the Int-Fit and Frail groups (*p* > 0. 05) but distinctly separated the Fit group (*p* < 0. 05). This result offers clear clinical insights, suggesting these risk groups can be managed as a unified high-risk category, simplifying decisions and improving clinical precision.

### 3.6. Discriminative performance of the Hemo-IMWG GA

To evaluate the discriminative performance of the models, we assessed the predictive ability of IMWG GA, HS, and Hemo-IMWG GA for grade ≥3 toxic events during the 1st to 4th chemotherapy cycles, examining AUC trends in both static and dynamic assessments.

The results showed that for total, non-hematological, and hematological toxicities, dynamic assessments (Table 5) outperformed static assessments (Supplementary Table S4). Specifically, in dynamic assessments, the Hemo-IMWG GA model consistently demonstrated superior discriminatory performance in predicting total toxicity (AUC 0.619–0.646; C-index 0.615, 95% CI: 0.571–0.658) and non-hematological toxicity (AUC 0.600–0.618; C-index 0.605, 95% CI: 0.557–0.651) compared to IMWG GA and HS in each cycle, indicating that the integrated model is more effective in recognizing frailty in these two areas than when used individually. For hematological toxicity, the Hemo-IMWG GA model still outperformed IMWG GA (C-index: 0.687 vs. 0.600). However, HS alone demonstrated the highest discriminative ability (AUC 0.686–0.697; C-index 0.717, 95% CI: 0.659–0.775).

**Table 5.** Discriminative performance of frailty models in dynamic evaluation.

Frailty Model	AE Category	AUC for 1-Cycle	AUC for 2-Cycle	AUC for 3-Cycle	AUC for 4-Cycle	C-index (Original)	C-index (Bootstrap)	95% CI (Bootstrap)
HS	Total	0.595	0.619	0.616	0.608	0.614	0.614	(0.573–0.655)
	Non-Hem	0.549	0.572	0.568	0.559	0.590	0.589	(0.546–0.633)
	Hem	0.696	0.686	0.697	0.687	0.719	0.717	(0.659–0.775)
IMWG GA	Total	0.589	0.604	0.606	0.596	0.581	0.580	(0.538–0.624)
	Non-Hem	0.599	0.607	0.606	0.595	0.590	0.590	(0.543–0.641)
	Hem	0.576	0.596	0.603	0.598	0.599	0.600	(0.528–0.669)
Hemo-IMWG GA	Total	0.619	0.646	0.640	0.640	0.615	0.615	(0.571–0.658)
	Non-Hem	0.600	0.618	0.614	0.611	0.606	0.605	(0.557–0.651)
	Hem	0.664	0.675	0.678	0.672	0.686	0.687	(0.630–0.740)

AE Categories: Total, total AEs; Non-Hem, non-hematological AEs; Hem, hematological AEs.

Following bootstrap correction (1,000 resamples) for all models, the differences between the corrected and original C-index values were all <0.002, indicating no significant overfitting and good overall model stability.

NRI analysis confirmed the additive value of HS integration, particularly for hematological AEs (NRI = 0.120), with smaller gains for total toxicity (NRI = 0.061) and minimal improvement for non-hematological AEs (NRI = 0.018) (Supplementary Table S5). However, DCA showed that the net clinical benefit of using Hemo-IMWG GA as a standalone decision tool was modest, with slight advantage over IMWG GA and HS for total AEs across clinically relevant thresholds (20–40%), but negligible benefit for non-hematological AEs and no apparent advantage for hematological AEs (Supplementary Figure S1).

## 4. Discussion

This study explores a more accurate dynamic evaluation strategy for chemotherapy tolerance in multiple myeloma (MM) patients. Integrating IMWG GA with HS, we developed the Hemo-IMWG GA, enhancing prediction accuracy for chemotherapy tolerance, especially for hematological AEs and interpretation of the Int-Fit group. Our results support the clinical application of frailty assessments, advancing towards a more dynamic and individualized management framework.

We noted dynamic changes in chemotherapy AEs among MM patients, with non-hematological AEs initially predominant, followed by hematological AEs in later treatment stages. Chemotherapy tolerance also varied dynamically across treatment cycles, a finding supported by several studies [5,15,16]. Notably, our analysis further demonstrated that dynamic assessments based on IMWG GA and HS outperformed static evaluations in terms of AE incidence, risk estimation, and model discrimination. This reinforces that static assessments fail to reflect real-time shifts in tolerance, underscoring the need for dynamic assessments. Currently, no consensus exists on the timing for these assessments [5,15–17], and the differences observed in our study suggest the need for flexible, periodic, or event-driven frailty assessments [18], thus aligning more closely with patients’ changing conditions.

The limitations of IMWG GA in stratifying hematological AEs arise from inherent design flaws, noted in early population studies and later validations [3,4]. Conversely, HS effectively differentiates hematological AEs (Fit vs Frail, HR = 9.91, *p* < 0. 001) and offers valuable insights into non-hematological AEs. Derived from routine blood tests, HS is highly accessible and holds significant clinical potential. Research shows that prolonged use of anti-myeloma drugs, like immunomodulatory drugs and cyclophosphamide, significantly damages bone marrow [19–21]. Our study found that hematological AEs escalated in later stages of induction therapy. Platelets, sensitive markers of bone marrow suppression [22,23], detect marrow damage within 7–10 days post-stem cell injury, typically reverting to baseline by day 28. Due to the dynamic nature of hematological AEs, periodic evaluation of HS is essential for accurate prediction of chemotherapy tolerance. Although HS may be influenced by disease burden, its dual attributes—baseline frailty shaped by tumor burden and sensitivity to treatment-induced hematological AEs—capture key features essential for assessing chemotherapy tolerance. Nevertheless, HS alone is limited in stratifying non-hematological AEs, necessitating a comprehensive assessment approach.

Recognizing that IMWG GA and HS excel at predicting non-hematological and hematological AEs respectively, with minimal overlap in the Frail group, we synthesized both tools into the Hemo-IMWG GA. The Hemo-IMWG GA integrates functional and hematopoietic dimensions, enhancing risk stratification and offering a comprehensive frailty evaluation for MM patients, notably addressing the limitations of IMWG GA in hematological AE prediction. This bi-dimensional strategy provides a more comprehensive framework for frailty assessment in clinical settings.

During model evaluation, Hemo-IMWG GA showed superior discrimination for total and non-hematological AEs, with higher AUC and C-index than IMWG GA and HS, underscoring its multidimensional value. For hematological AEs, it outperformed IMWG GA but remained inferior to HS, reflecting the latter’s sensitivity to hematopoietic changes. NRI analysis further highlighted the benefit of HS integration, with the largest improvement in hematological AEs (NRI = 0.120), while gains for other endpoints were minor. However, DCA showed that the net clinical benefit of using Hemo-IMWG GA as a standalone decision tool was modest, with only slight advantage for overall AEs and minimal benefit for specific AE types. This likely reflects real-world practice, where toxicity-driven interventions are often limited to temporary treatment interruptions and supportive care, rather than definitive dose reduction or regimen change, thereby diminishing the incremental impact of model-based predictions on long-term risk mitigation. Additionally, limited availability of alternative regimens and a lack of systematic incorporation of frailty assessments into treatment adjustment may further attenuate the potential clinical utility of such models. Despite these limitations, Hemo-IMWG GA remains clinically relevant as a tool for risk stratification rather than deterministic decision-making. Its strength lies in identifying high-risk patients and may help guide proactive monitoring or early supportive interventions, particularly within dynamic assessment frameworks, rather than serving as a standalone trigger for treatment modification.

The Hemo-IMWG GA enhances risk stratification and effectively resolves the ‘gray zone’ issue in the Int-Fit group. Interpreting the Int-Fit group in three-tier frailty assessment tools is frequently problematic. Comprising 20–60% of study populations, this group restricts the efficacy of frailty tools in clinical decisions [16,24–27]. Although binary classification of frailty status (e. g., simplified frailty scores [28]) may circumvent the gray zone, it potentially compromises the model’s reliability [29]. The Hemo-IMWG GA aligns the Int-Fit group’s risk closer to the Frail group, suggesting it be treated as ‘potentially frail’ and managed akin to the Frail group. This method offers clearer clinical guidance and improved interpretability. Comparable findings have been observed with the TM frailty score [4]. Although this study did not perform prognostic analyses, existing data support the inclusion of the Int-Fit group in the Frail group intervention pathway.

Unlike most studies that examine frailty’s relationship with long-term outcomes (PFS/OS) [30], this study concentrates on how frailty affects chemotherapy tolerance. Long-term outcomes, influenced by diverse factors like treatment regimens and genetic backgrounds [31,32], may see diminished predictive reliability over time. Conversely, frailty has a direct, measurable impact on chemotherapy tolerance: worsening frailty correlates with increased AEs and early mortality [33–35], yet addressing frailty can enhance tolerance, minimize AEs, and lower mortality rates [35,36]. Additionally, tolerance assessments offer clinicians timely and precise guidance to adapt treatment plans effectively, ensuring patients can handle the current treatment intensity. In clinical decision-making, it’s crucial to prioritize assessing a patient’s tolerance within each treatment cycle, determining if they can sustain standard intensity or require dose adjustments or additional support [37]. Considering these factors, we prioritized examining the relationship between chemotherapy tolerance and frailty status over prognosis as an endpoint.

This study has several limitations. Firstly, as a single-center, retrospective study with a limited sample size (*n* = 111) and a relatively young cohort (median age 61 years), there may be selection bias, which restricts the generalizability of our findings, especially to older or more frail populations. Although internal validation using bootstrap resampling demonstrated good model stability, external validation in larger, multi-center cohorts remains essential. Secondly, the retrospective design resulted in missing key data, including some cytogenetic information and grade 1–2 adverse events, and did not account for treatment-related modifications such as dose adjustments, delays, or supportive care (e.g. G-CSF use), all of which could influence toxicity risk. This limitation may also help explain the modest net clinical benefit observed in decision curve analysis. Future prospective studies should systematically collect these data and incorporate real-world treatment modifications (e.g. dose reductions, delays, supportive care) into analytic models to minimize confounding and better reflect clinical practice. Thirdly, although our dynamic evaluation strategy is promising, its clinical implementation and effect on treatment adaptation have not yet been tested in prospective interventional trials. Future studies should explore embedding this scoring tool within frailty-tailored treatment algorithms and dynamic monitoring frameworks to translate its predictive value into actionable clinical strategies and optimize chemotherapy tolerance and patient outcomes.

## 5. Conclusion

This study underscores the importance of dynamic frailty assessment for predicting chemotherapy tolerance in MM patients. We developed the Hemo-IMWG GA by integrating IMWG GA with HS, significantly improving predictions of chemotherapy-related AEs, particularly hematological toxicity. A dynamic evaluation of frailty that includes both functional and hematopoietic dimensions provides a thorough method for assessing chemotherapy tolerance. Our results indicate that the Hemo-IMWG GA is a practical and accessible tool that can guide personalized treatment decisions. Further research is needed to validate this score across larger cohorts and to assess its impact on enhancing personalized treatment strategies.

## Supplementary Material

Supplemental Material

## Data Availability

All data generated or analyzed during this study are included in this published article and its supplementary information files.

## References

[1] Puertas B, González-Calle V, Sobejano-Fuertes E, et al. Novel agents as main drivers for continued improvement in survival in multiple myeloma [J]. Cancers (Basel). 2023;15(5):1558. doi: 10.3390/cancers15051558. 36900349 PMC10000382

[2] Lipof JJ, Abdallah N, Lipe B. Personalized treatment of multiple myeloma in frail patients [J]. Curr Oncol Rep. 2024;26(7):744–753. doi: 10.1007/s11912-024-01545-2. 38761302

[3] Palumbo A, Bringhen S, Mateos MV, et al. Geriatric assessment predicts survival and toxicities in elderly myeloma patients: an International Myeloma Working Group report[J]. Blood. 2015;125(13):2068–2074. doi: 10.1182/blood-2014-12-615187. 25628469 PMC4375104

[4] Chen Y, Gu J, Huang B, et al. Development and validation of a chemotherapy tolerance prediction model for Chinese multiple myeloma patients: the TM frailty score[J]. Front Oncol. 2023;13:1103687. doi: 10.3389/fonc.2023.1103687. 36741003 PMC9895409

[5] Zhang Y, Liang X, Xu W, et al. Individualized dynamic frailty-tailored therapy (DynaFiT) in elderly patients with newly diagnosed multiple myeloma: a prospective study[J]. J Hematol Oncol. 2024;17(1):48. doi: 10.1186/s13045-024-01569-y. 38915117 PMC11197371

[6] Olivieri F, Sabbatinelli J, Bonfigli AR, et al. Routine laboratory parameters, including complete blood count, predict COVID-19 in-hospital mortality in geriatric patients[J]. Mech Ageing Dev. 2022;204:111674. doi: 10.1016/j.mad.2022.111674. 35421418 PMC8996472

[7] Al Saleh AS, Sidiqi MH, Dispenzieri A, et al. Hematopoietic score predicts outcomes in newly diagnosed multiple myeloma patients[J]. Am J Hematol. 2020;95(1):4–9. doi: 10.1002/ajh.25657. 31612526 PMC7377299

[8] Ratajczak MZ, Kucia M. Hematopoiesis and innate immunity: an inseparable couple for good and bad times, bound together by an hormetic relationship[J]. Leukemia. 2022;36(1):23–32. doi: 10.1038/s41375-021-01482-0. 34853440 PMC8727304

[9] Long J, Lai H, Huang Y, et al. Unraveling the pathogenesis of bone marrow hematopoietic injury and the therapeutic potential of natural products[J]. Pharmacol Res. 2025;212:107589. doi: 10.1016/j.phrs.2025.107589. 39778641

[10] Corona LP, Andrade FCD, Da Silva Alexandre T, et al. Higher hemoglobin levels are associated with better physical performance among older adults without anemia: a longitudinal analysis[J]. BMC Geriatr. 2022;22(1):233. doi: 10.1186/s12877-022-02937-4. 35313814 PMC8939094

[11] Green R, Dwyre DM. Evaluation of macrocytic anemias[J]. Semin Hematol. 2015;52(4):279–286. doi: 10.1053/j.seminhematol.2015.06.001. 26404440

[12] Lee JY, Choi H, Park JW, et al. Age-related changes in mean corpuscular volumes in patients without anaemia: an analysis of large-volume data from a single institute[J]. J Cell Mol Med. 2022;26(12):3548–3556. doi: 10.1111/jcmm.17397. 35599236 PMC9189337

[13] Sonneveld P, Avet-Loiseau H, Lonial S, et al. Treatment of multiple myeloma with high-risk cytogenetics: a consensus of the International Myeloma Working Group[J]. Blood. 2016;127(24):2955–2962. doi: 10.1182/blood-2016-01-631200. 27002115 PMC4920674

[14] Richardson PG, Perrot A, Mikhael J, et al. Allocation and validation of the second revision of the International Staging System in the ICARIA-MM and IKEMA studies[J]. Blood Cancer J. 2024;14(1):209. doi: 10.1038/s41408-024-01149-w. 39609425 PMC11605113

[15] Smits F, Groen K, Levin MD, et al. Beyond static measurements: dynamic frailty improves survival prediction in multiple myeloma[J]. Blood. 2025;145(5):543–546. doi: 10.1182/blood.2024025868. 39576961

[16] Mian H, Wildes TM, Vij R, et al. Dynamic frailty risk assessment among older adults with multiple myeloma: a population-based cohort study[J]. Blood Cancer J. 2023;13(1):76. doi: 10.1038/s41408-023-00843-5. 37164972 PMC10172354

[17] Cook G, Larocca A, Facon T, et al. Defining the vulnerable patient with myeloma-a frailty position paper of the European Myeloma Network[J]. Leukemia. 2020;34(9):2285–2294. doi: 10.1038/s41375-020-0918-6. 32555295 PMC7449877

[18] Clegg A, Young J, Iliffe S, et al. Frailty in elderly people[J]. Lancet. 2013;381(9868):752–762. doi: 10.1016/S0140-6736(12)62167-9. 23395245 PMC4098658

[19] Schoenbeck KL, Wildes TM. Updated perspectives on the management of multiple myeloma in older patients: focus on lenalidomide[J]. Clin Interv Aging. 2020;15:619–633. doi: 10.2147/CIA.S196087. 32440105 PMC7210019

[20] Mushtaq A, Kapoor V, Latif A, et al. Efficacy and toxicity profile of carfilzomib based regimens for treatment of multiple myeloma: a systematic review[J]. Crit Rev Oncol Hematol. 2018;125:1–11. doi: 10.1016/j.critrevonc.2018.02.008. 29650268 PMC5901887

[21] Ba Y, Shi Y, Jiang W, et al. Current management of chemotherapy-induced neutropenia in adults: key points and new challenges: committee of Neoplastic Supportive-Care (CONS), China Anti-Cancer Association Committee of Clinical Chemotherapy, China Anti-Cancer Association[J]. Cancer Biol Med. 2020;17(4):896–909. doi: 10.20892/j.issn.2095-3941.2020.0069. 33299642 PMC7721096

[22] Soff G, Leader A, Al-Samkari H, et al. Management of chemotherapy-induced thrombocytopenia: guidance from the ISTH Subcommittee on Hemostasis and Malignancy[J]. J Thromb Haemost. 2024;22(1):53–60. doi: 10.1016/j.jtha.2023.09.031. 37827380

[23] Kuter DJ. Treatment of chemotherapy-induced thrombocytopenia in patients with non-hematologic malignancies[J]. Haematologica. 2022;107(6):1243–1263. doi: 10.3324/haematol.2021.279512. 35642485 PMC9152964

[24] Murugappan MN, King-Kallimanis BL, Bhatnagar V, et al. Patient-reported frailty phenotype (PRFP) vs. International Myeloma Working Group frailty index (IMWG FI) proxy: a comparison between two approaches to measuring frailty[J]. J Geriatr Oncol. 2024;15(2):101681. doi: 10.1016/j.jgo.2023.101681. 38104480

[25] Gahagan A, Maheshwari S, Rangarajan S, et al. Evaluating concordance between International Myeloma Working Group (IMWG) frailty score and simplified frailty scale among older adults with multiple myeloma[J]. J Geriatr Oncol. 2024;15(8):102051. doi: 10.1016/j.jgo.2024.102051. 39241344

[26] Abdallah N, Dizona P, Kumar A, et al. Cumulative deficits frailty index and relationship status predict survival in multiple myeloma[J]. Blood Adv. 2025;9(5):1137–1146. doi: 10.1182/bloodadvances.2024014624. 39693516 PMC11914168

[27] Schoeller K, Ihorst G, Scheubeck S, et al. The Revised Myeloma Comorbidity Index (R-MCI) as a promising approach for predicting overall (OS)- and progression-free (PFS) survival and optimizing therapy strategies in Multiple Myeloma (MM) Patients (pts) - Comparative analysis of 5 Comorbidity Indices (CI), including retro- and prospective applicability[J]. Blood. 2019;134(Supplement_1):3474–3474. doi: 10.1182/blood-2019-127030.

[28] Facon T, Dimopoulos MA, Meuleman N, et al. A simplified frailty scale predicts outcomes in transplant-ineligible patients with newly diagnosed multiple myeloma treated in the FIRST (MM-020) trial[J]. Leukemia. 2020;34(1):224–233. doi: 10.1038/s41375-019-0539-0. 31427722 PMC7214253

[29] Groen K, Smits F, Nasserinejad K, et al. Assessing frailty in myeloma: the pursuit of simplicity may sacrifice precision of predicting clinical outcomes[J]. Hemasphere. 2024;8(7):e85. doi: 10.1002/hem3.85. 38966208 PMC11223651

[30] Mian H, Mccurdy A, Giri S, et al. The prevalence and outcomes of frail older adults in clinical trials in multiple myeloma: a systematic review[J]. Blood Cancer J. 2023;13(1):6. doi: 10.1038/s41408-022-00779-2. 36599867 PMC9813365

[31] Xu L, Wang X, Pan X, et al. Education level as a predictor of survival in patients with multiple myeloma[J]. BMC Cancer. 2020;20(1):737. doi: 10.1186/s12885-020-07178-5. 32770980 PMC7414648

[32] Rajkumar SV. Multiple myeloma: 2024 update on diagnosis, risk-stratification, and management[J]. Am J Hematol. 2024;99(9):1802–1824. doi: 10.1002/ajh.27422. 38943315 PMC11404783

[33] Handforth C, Clegg A, Young C, et al. The prevalence and outcomes of frailty in older cancer patients: a systematic review[J]. Ann Oncol. 2015;26(6):1091–1101. doi: 10.1093/annonc/mdu540. 25403592

[34] Zhang F, Yan Y, Ge C. Frailty as a predictor of adverse outcomes in patients with gastric cancer: a systematic review and meta-analysis of 75,357 patients[J]. Ageing Res Rev. 2024;101:102528. doi: 10.1016/j.arr.2024.102528. 39362340

[35] Ríos-Tamayo R, Lecumberri R, Cibeira MT, et al. A simple frailty score predicts survival and early mortality in systemic AL amyloidosis[J]. Cancers (Basel). 2024;16(9):1689. doi: 10.3390/cancers16091689. 38730641 PMC11083900

[36] Goede V. Frailty and cancer: current perspectives on assessment and monitoring[J]. Clin Interv Aging. 2023;18:505–521. doi: 10.2147/CIA.S365494. 37013130 PMC10066705

[37] Peipert JD, Smith ML, EVOLV Study Team. Reconsidering tolerability of cancer treatments: opportunities to focus on the patient[J]. Support Care Cancer. 2022;30(5):3661–3663. doi: 10.1007/s00520-021-06700-0. 35013779 PMC9276550

