# Supplementary material for: Combining IMWG GA and hematopoietic score to optimize the evaluation of dynamic chemotherapy tolerance in multiple myeloma
Source: Ann Med. 2025 Oct 14;57(1):2558127. doi: 10.1080/07853890.2025.2558127 (PMC12523449; doi:10.1080/07853890.2025.2558127)
Supplement: Supplemental Material [file IANN_A_2558127_SM4828.docx]

## Supplementary Table 1. Distribution and Classification of Grade ≥3 AEs During Induction Chemotherapy

| **Category** | | | **N** | **Category (%)** | **Total (%)** |
| --- | --- | --- | --- | --- | --- |
| **Non-Hematological AEs** | | | **210** | **100%** | **67.96%** |
|  | **Infectious AEs** | | **150** | **71.43%** | **48.54%** |
|  |  | Abdominal | 6 | 2.86% | 1.94% |
|  |  | Bloodstream Infection | 6 | 2.86% | 1.94% |
|  |  | Catheter-related Infection | 1 | 0.48% | 0.32% |
|  |  | Pulmonary | 112 | 53.33% | 36.25% |
|  |  | Urinary Tract Infection | 5 | 2.38% | 1.62% |
|  |  | Upper Respiratory Tract Infection | 6 | 2.86% | 1.94% |
|  |  | Skin and Soft Tissue | 5 | 2.38% | 1.62% |
|  |  | Uncertain Position | 3 | 1.43% | 0.97% |
|  |  | Herpes Zoster | 6 | 2.86% | 1.94% |
|  | **Non-Infectious AEs** | | **60** | **28.57%** | **19.42%** |
|  |  | Atrial Fibrillation | 1 | 0.48% | 0.32% |
|  |  | Heart Failure | 21 | 10.00% | 6.80% |
|  |  | gastrointestinal Intolerance | 1 | 0.48% | 0.32% |
|  |  | Diarrhea | 15 | 7.14% | 4.85% |
|  |  | Epilepsy | 1 | 0.48% | 0.32% |
|  |  | Muscle Dysfunction | 1 | 0.48% | 0.32% |
|  |  | Peripheral Neuropathy | 9 | 4.29% | 2.91% |
|  |  | Rash | 8 | 3.81% | 2.59% |
|  |  | Anaphylactic Shock | 1 | 0.48% | 0.32% |
|  |  | Death | 2 | 0.95% | 0.65% |
| **Hematological AEs** | | | **99** | **100.00%** | **32.04%** |
|  |  | Anemia | 24 | 24.24% | 7.77% |
|  |  | Leukopenia | 15 | 15.15% | 4.85% |
|  |  | Thrombocytopenia | 8 | 8.08% | 2.59% |
|  |  | Lymphopenia | 22 | 22.22% | 7.12% |
|  |  | Neutropenia | 27 | 27.27% | 8.74% |
|  |  | Aminotransferase | 3 | 3.03% | 0.97% |
| **Combined Total** | |  | **309** |  | **100.00%** |

## Supplementary Table 2. Dynamic Changes in AEs Across Chemotherapy Cycles

| **AE Category** | **Cycle 1** | **Cycle 2** | **Cycle 3** | **Cycle 4** | **Cycle 5** | **Cycle 6** |
| --- | --- | --- | --- | --- | --- | --- |
| **Total AEs** | 61 (100%) | 74 (100%) | 53 (100%) | 61 (100%) | 33 (100%) | 27 (100%) |
| **Non-Hematological AEs** | 58 (95.1%) | 54 (73.0%) | 32 (60.4%) | 35 (57.4%) | 16 (48.5%) | 15 (55.6%) |
| **Hematological AEs** | 3 (4.9%) | 20 (27.0%) | 21 (39.6%) | 26 (42.6%) | 17 (51.5%) | 12 (44.4%) |

## Supplementary Table 3. Dynamic Changes in HS and IMWG GA Across Chemotherapy Cycles

| **Frailty Model** | **Subgroup** | **Cycle 1** | **Cycle 2** | **Cycle 3** | **Cycle 4** | **Cycle 5** | **Cycle 6** |
| --- | --- | --- | --- | --- | --- | --- | --- |
| **HS** | Fit | 30 (27.0%) | 31 (28.4%) | 35 (36.1%) | 38 (41.8%) | 37 (49.3%) | 31 (50.0%) |
|  | Int-Fit | 39 (35.1%) | 42 (38.5%) | 40 (41.2%) | 32 (35.2%) | 20 (26.7%) | 19 (30.6%) |
|  | Frail | 42 (37.8%) | 36 (33.0%) | 22 (22.7%) | 21 (23.1%) | 18 (24.0%) | 12 (19.4%) |
| **IMWG GA** | Fit | 46 (41.4%) | 45 (41.3%) | 49 (50.5%) | 48 (52.7%) | 39 (52.0%) | 33 (53.2%) |
|  | Int-Fit | 24 (21.6%) | 33 (30.3%) | 24 (24.7%) | 19 (20.9%) | 19 (25.3%) | 12 (19.4%) |
|  | Frail | 41 (36.9%) | 31 (28.4%) | 24 (24.7%) | 24 (26.4%) | 17 (22.7%) | 17 (27.4%) |

## Supplementary Table 4. Discriminative Performance of Frailty Models in Static Evaluation

| **Frailty Model** | **AE Category** | **AUC for 1-Cycle** | **AUC for 2-Cycle** | **AUC for 3-Cycle** | **AUC for 4-Cycle** | **C-index (Original)** | **C-index (Bootstrap)** | **95% CI (Bootstrap)** |
| --- | --- | --- | --- | --- | --- | --- | --- | --- |
| **HS** | Total | 0.504 | 0.512 | 0.510 | 0.513 | 0.517 | 0.517 | (0.475 – 0.563) |
|  | Non-Hem | 0.469 | 0.478 | 0.473 | 0.475 | 0.494 | 0.495 | (0.450 – 0.543) |
|  | Hem | 0.620 | 0.631 | 0.642 | 0.651 | 0.625 | 0.625 | (0.558 – 0.687) |
| **IMWG GA** | Total | 0.554 | 0.553 | 0.558 | 0.569 | 0.543 | 0.542 | (0.498 – 0.585) |
|  | Non-Hem | 0.565 | 0.560 | 0.561 | 0.574 | 0.552 | 0.553 | (0.504 – 0.598) |
|  | Hem | 0.552 | 0.560 | 0.572 | 0.574 | 0.557 | 0.557 | (0.492 – 0.624) |
| **Hemo-IMWG GA** | Total | 0.545 | 0.548 | 0.552 | 0.566 | 0.539 | 0.540 | (0.495 – 0.587) |
|  | Non-Hem | 0.533 | 0.534 | 0.532 | 0.546 | 0.534 | 0.533 | (0.483 – 0.581) |
|  | Hem | 0.609 | 0.624 | 0.642 | 0.650 | 0.609 | 0.610 | (0.551 – 0.669) |
| AE Categories: Total, total AEs; Non-Hem, non-hematological AEs; Hem, hematological AEs. | | | | | | | | |

## Supplementary Table 5. Net Reclassification Improvement (NRI) of Frailty Models

| **AE Category** | **NRI** | **Improved Events** | **Worsened Events** | **Improved Non-Events** | **Worsened Non-Events** |
| --- | --- | --- | --- | --- | --- |
| **Total** | 0.061 | 0.108 | 0.036 | 0.074 | 0.086 |
| **Non-Hem** | 0.018 | 0.092 | 0.046 | 0.066 | 0.095 |
| **Hem** | 0.120 | 0.138 | 0.000 | 0.069 | 0.088 |
| AE Categories: Total, total AEs; Non-Hem, non-hematological AEs; Hem, hematological AEs. | | | | | |

| 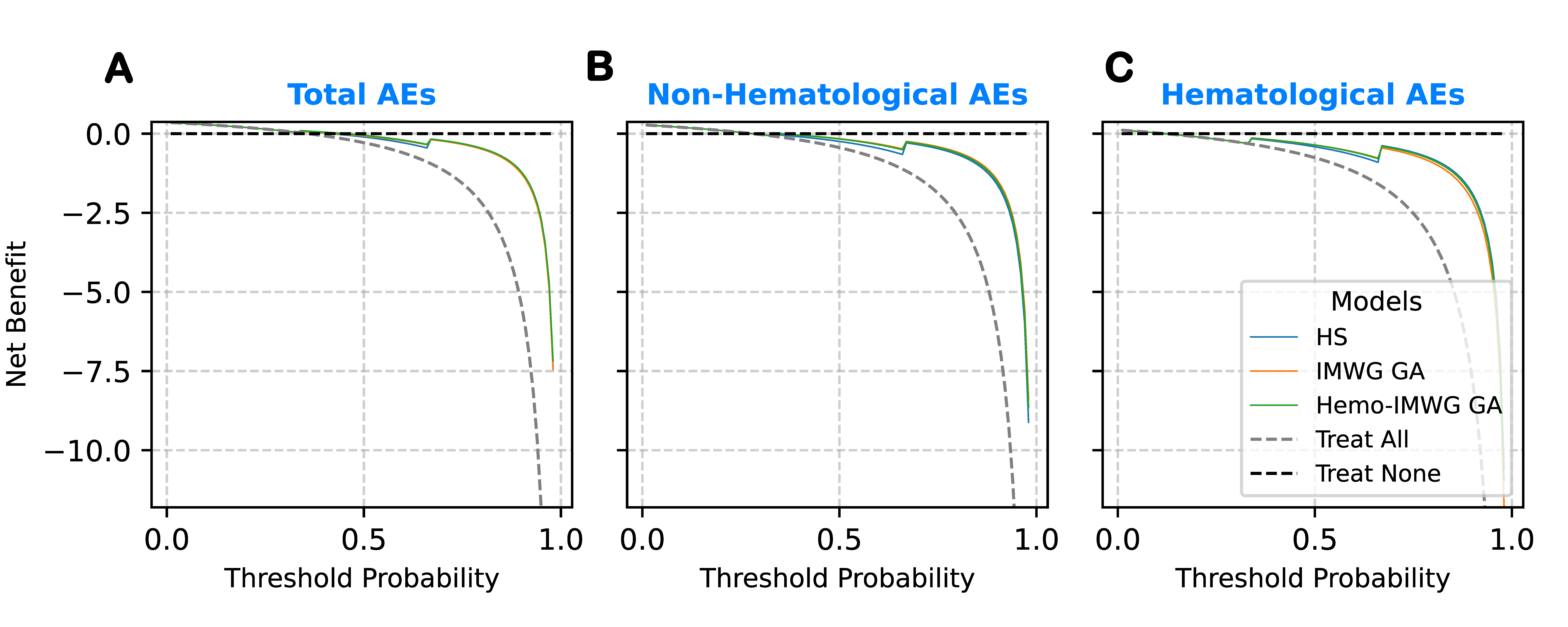 |
| --- |
| **Supplementary Fig. 1**: Decision Curve Analysis of Frailty Models for Predicting Grade ≥3 AEs  The decision curve analysis (DCA) evaluates the net clinical benefit of IMWG GA, HS, and Hemo-IMWG GA for predicting grade ≥ 3 AEs: (A) total AEs, (B) non-hematological AEs, and (C) hematological AEs. The Hemo-IMWG GA yielded a slightly higher net benefit for total AEs within clinically relevant threshold probabilities (20–40%) compared with IMWG GA and HS. However, no clear advantage was observed for non-hematological or hematological AEs. |
